# Key Decision Makers and Actors in Selected Newborn Care Practices: A Community-Based Survey in Northern Uganda

**DOI:** 10.3390/ijerph16101723

**Published:** 2019-05-16

**Authors:** David Mukunya, Victoria Nankabirwa, Grace Ndeezi, Josephine Tumuhamye, Justin Bruno Tongun, Samuel Kizito, Agnes Napyo, Vincentina Achora, Beatrice Odongkara, Agnes Anna Arach, Thorkild Tylleskar, James K Tumwine

**Affiliations:** 1 Center for International Health, University of Bergen, 7804 Bergen, Norway; nankabirwav@gmail.com (V.N.); tphynne@gmail.com (J.T.); Thorkild.Tylleskar@uib.no (T.T.); 2 Department of Epidemiology and Biostatistics, School of Public Health, Makerere University, 7062 Kampala, Uganda; 3 Department of Pediatrics and Child Health, Makerere University, 7062 Kampala, Uganda; gndeezi@gmail.com (G.N.); kabaleimc@gmail.com (J.K.T.); 4 Department of Pediatrics, University of Juba, 82 Juba, South Sudan; tongunmafi@gmail.com; 5 Clinical Epidemiology Unit, Makerere University, 7062 Kampala, Uganda; somekizito@yahoo.com; 6 Department of Public Health, Busitema University, 236 Tororo, Uganda; napyoagnes@gmail.com; 7 Department of Obstetrics and Gynecology, University of Gulu, 166 Gulu, Uganda; vincentachora@gmail.com; 8 Department of Pediatrics, University of Gulu, 166 Gulu, Uganda; beachristo2003@gmail.com; 9 Department of Nursing, Lira University, 1035 Lira, Uganda; Arachaa2000@gmail.com

**Keywords:** newborn, neonatal, implementation, influencers, actors, decision-making, Uganda, post-conflict, behavior

## Abstract

Knowledge of key decision makers and actors in newborn care is necessary to ensure that health interventions are targeted at the right people. This was a cross-sectional study carried out in Lira district, Northern Uganda. Multivariable logistic regression was used to determine factors associated with mothers being the key decision maker regarding where to give birth from and when to initiate breastfeeding. Fathers were the key decision makers on the place of birth (54.3%, *n* = 505) and on whether to seek care for a sick newborn child (47.7%, *n* = 92). Grandmothers most commonly bathed the baby immediately after birth (55.5%, *n* = 516), whereas mothers and health workers were common decision makers regarding breastfeeding initiation. Predictors for a mother being the key decision maker on the place of birth included: Mother having a secondary education (AOR 1.9: 95% C.I (1.0–3.6)) and mother being formally employed (AOR 2.0: 95% (1.5–2.9)). Mothers, fathers, grandmothers, health-workers, and traditional birth attendants were the most influential in the selected newborn care practices. Programs that aim to promote newborn care need to involve husbands, grandmothers, and health workers in addition to mothers.

## 1. Introduction

Uganda has unacceptably high under-5-year child mortality; 62.4 deaths per 1000 live births compared to the worldwide estimate of 38.4 deaths per 1000 live births [1]. A large proportion of deaths under the age of 5 years occur in the newborn/neonatal period (first 28 days of life), with 22.3 newborn deaths per 1000 live births occurring in Uganda compared to the global estimate of 16.7 deaths per 1000 [1]. Most of these deaths are preventable, and low-cost interventions that can significantly reduce them exist [2,3,4]. It is estimated that existing low-cost interventions can reduce newborn deaths by 72% [3]. Nonetheless, the scale-up of these low-cost interventions in sub-Saharan Africa has been both poor and inequitable [5]. One of the main challenges to scaling up these interventions has been the under-recognition of the behavioral and sociocultural aspects of newborn care practices [4].

One under-recognized sociocultural aspect of newborn care is the primary caretakers of newborns in the perinatal period [4]. Traditionally, it has been thought that mothers are the key decision makers in the perinatal period [6,7]. However, since mothers are often (or perceived to be) exhausted in the immediate post-partum period, and may be inexperienced in newborn care (especially first-time mothers), older relatives often take over the role of caring for mothers and newborns [8]. Antenatal education usually promotes recommended care practices like newborn care, timely health care seeking, optimal breastfeeding, and also educates mothers on danger signs in pregnancy [9]. The education is usually provided by health workers, at health facilities, and mainly targets the pregnant women, and not the significant others [10]. As a result, the persons who take care of newborns seldom receive antenatal education concerning recommended practices in newborn care [11]. To design effective interventions that promote behavioral change in newborn care, it is important to identify the key decision makers and actors in newborn care [4]. Whereas multiple players could be responsible for various actions in the newborn period, our experience with the study population showed that there was often a key decision maker or an actor, who was ultimately responsible for the action. Previous research has highlighted some decision makers and actors, but these have largely been obtained in qualitative studies [8,12] and hence cannot estimate the magnitude, the relative contribution of different players, and the generalizability to wider populations.

In this study, we aimed to quantitatively determine key decision makers and actors in newborn care among mothers with children under the age of 2 years in the Lira district, Northern Uganda.

## 2. Materials and Methods

### 2.1. Study Design

This was a cross-sectional study that involved mothers who had a child below the age of 2 years.

#### 2.1.1. Study Setting

The study was carried out between August and November 2016 in the Lira district, Northern Uganda. The district has 13 subcounties, 1 municipality, and 751 villages [13,14]. The majority of the population is ethnic Langi, and the predominant language spoken is Lango. The study was carried out in 3 subcounties in Northern Uganda: Aromo, Agali, and the Lira municipality. These subcounties were chosen because they had poor maternal and child health indicators and were ideal for an interventional study designed to increase facility-based births, and to improve breastfeeding practices.

#### 2.1.2. Sampling

All villages in the 3 selected subcounties were listed, and 30 villages were selected by probability proportional to size. A sampling frame of all households in the village obtained from the village leaders was used to randomly select a household to begin with in each village. After identifying the first household, the next household was chosen based on proximity to the first. The nearest household was defined as one whose door was closest to the prior household. Only one mother–child pair would be chosen from each household. This process would continue until 31 mother–child pairs had been interviewed in each village. If the mother had more than one child born within the previous 2 years, questions were addressed to the most recent pregnancy and therefore the youngest child. If the mother had twins, questions were aimed at the younger twin.

#### 2.1.3. Study Participants

We recruited mothers with children born within the last 2 years. Mothers were recruited only if they were residents in the respective village as determined by the village leadership and were mentally and physically able to complete the interview.

#### 2.1.4. Study Procedures

Data collection was carried out with the help of 30 research assistants who were recruited from Lira district and were well conversant with the local languages. Data were collected using Open Data Kit (ODK, https://opendatakit.org/) software installed on mobile phones. The questionnaires were translated to the local language and back-translated to English for accuracy. Village health team members and local area leaders acted as our guides. Households whose members were absent at the time of the visit were revisited later on the same day, prior to exiting the village. Those that were still absent were revisited the following day and, if not found, were declared missing and replaced. Interviews were carried out at the mothers’ homesteads, preferably at a private place away from distraction, e.g., under a tree at the periphery of the compound. The interviews lasted between 45 and 60 min. The questions asked were close-ended questions.

### 2.2. Variables

To determine the key decision makers and actors in newborn care, mothers were asked the following questions regarding their most recent birth experience: Who mainly decided where you should give birth? Who dried your baby immediately after birth? Who first bathed your baby? Who mainly decided when you initiated breastfeeding? Who mainly decided what you did with the initial breast milk? Who mainly decided whether or not you practiced bottle-feeding? Who applied substances to the umbilical cord of your child immediately after birth? Who mainly decided whether or not to seek care for your sick newborn? (Addressed to only those who reported having had a sick newborn). We defined key decision makers and actors as the most frequently cited decision maker or actor, and common decision makers and actors as the two most frequently cited decision makers and actors. The newborn care practices studied were obtained from lists of recommended newborn care practices [15,16,17,18]. Mothers were classified as formally employed if they personally earned money through some activities. Socioeconomic status quintiles were calculated from an asset-based wealth index using principal component analysis. Other variables collected included: maternal age, maternal education, paternal education, marital status, parity, residence, maternal employment, and place of birth. These were collected to offer a general description of the study participants, and to act as explanatory variables or confounders in the multivariable analysis. We classified place of birth as a newborn care practice with an understanding that newborn care begins during birth, and therefore, the choice of the place of childbirth is a distal newborn care decision.

### 2.3. Sample Size Estimation

The sample size was calculated for the primary objective of this study. A total of 930 mothers were enrolled in the study. This was calculated by Open-epi software (www.openepi.com), assuming that a proportion of 50% of women decide where they should give birth. We also assumed precision of 5% and a design effect of 2. This gave us a sample size of 768 participants. Assuming a nonresponse of 15%, we came up with a minimum sample size of 904 mothers. To achieve an equal enumeration in all the 30 villages, we purposed to enroll at least 31 mothers from each village. The sample size calculated was sufficient to determine the different proportions of decision makers, influencers, and actors, assuming that 50% of mothers were the key decision makers concerning all practices.

### 2.4. Data Analysis

The analysis strategy considered the fact that we used multistage sampling. Descriptive variables were presented as means and proportions for continuous and categorical variables, respectively. Factors with a *p*-value <0.25 at bi-variable analysis were considered for the multivariable model. Collinearity was assessed, and factors were considered to be strongly collinear if their variance inflation factor was greater than ten. In cases where factors were found to be collinear, the factor with a stronger scientific plausibility was retained in the model. Our final multivariable models consisted of factors with strong scientific plausibility, factors with significant associations at bi-variable analysis, and factors which changed the measure of association of any covariate in the intermediate models by greater than 10%. As stated before, this study was primarily designed to determine the prevalence of health facility births, and breastfeeding practices in the study area in order to inform the design and interpretation of a cluster randomized controlled study in the area. We, therefore, present the multivariable analysis of only the key decision makers regarding health facility births and breastfeeding initiation, as these were of primary interest to the interpretation of the intervention study. We stratified our results by place of residence (rural versus urban), place of birth (health facility versus home), and parity. We used these strata because qualitative literature suggested there could be differences in key decision makers in these strata [8,12,19,20,21,22].

### 2.5. Ethics

We obtained ethical approval from the Makerere University School of Medicine Research and Ethics committee (SOMREC number: 2015-121) and from the Uganda National Council of Science and Technology. District and local area leaders gave us permission to perform the study. Written informed consent was obtained from the participants prior to participation in the study. For participants who could not write, a thumbprint was obtained.

## 3. Results

### 3.1. Descriptive Characteristics

A total of 930 mother–child pairs were considered for the analysis. The response rate was 93%. The majority (>95%) of non-respondents were mothers who were absent from their homestead. The mean child age was 11.2 months, with a standard deviation of 7.7 months. The mean maternal age was 25.8, with a standard deviation of 5.9 years. The majority of mothers (729, 78.4%) and fathers (524, 61.4%) had less than 7 years of formal education. The rest of the participant characteristics are presented in Table 1.

### 3.2. Decision Makers and Actors in Newborn Care

Fathers most commonly decided on where a mother gave birth (54.3%, *n* = 505), but the mothers (31.1%, *n* = 289) were also common decision makers. Fathers also most commonly decided on whether to seek care for a sick newborn child (47.7%, *n* = 92). Grandmothers most commonly bathed the baby immediately after birth (55.5%, *n* = 516), whereas mothers most commonly decided on when to initiate breastfeeding (53.7%, *n* = 499) and on whether to bottle-feed (73.6%, *n* = 684) (Table 2). Health workers were also commonly cited in the decision to initiate breastfeeding and to practice bottle-feeding. Traditional birth attendants were most implicated in applying substances to the umbilical cords of newborns immediately after birth (Table 2). The distribution of key decision makers in the subgroup analysis (rural residence, first-time mothers, and home births) was similar to that of the overall population. A notable difference in the subgroup analysis was that grandmothers were more involved with first-time mothers (Table 3). Table A1 in the Appendix A provides a detailed breakdown of the main practices and actors studied.

### 3.3. Factors Associated with the Mother Being the Key Decision Maker

At multivariable analysis, the factors associated with the mother being the key decision maker for where she gave birth included: Mother having a secondary education compared to no education (adjusted odds ratio, AOR 1.9: (95% CI 1.0, 3.6)), mother having parity of 4 or more compared to one (AOR 2.1: (95% CI 1.2, 3.8)), and mother being employed (AOR 2.0: (95% 1.5, 2.9)) (Table 4). Mothers from households that belonged to the wealthiest quintile were less likely to be mainly responsible for where they gave birth from as compared to mothers from households in the poorest quintile (AOR 0.44: (95% CI 0.25, 0.76)) (Table 4).

Regarding the decision to initiate breastfeeding, older mothers were more likely to decide on when to initiate breastfeeding (AOR 1.3: (95% 0.58,3.1)), whereas mothers in rural areas were less likely to make this decision (AOR 0.72: (95% CI 0.52, 0.99)) (Table 5).

## 4. Discussion

This study showed that mothers, fathers, grandmothers, health workers, and traditional birth attendants were the key decision makers and actors in newborn care, albeit for different practices. This finding is not surprising and has been reported by qualitative findings in other African countries [4,8,23]. This study contributes to the existing literature by adding a quantitative dimension to those findings.

We found that in the majority of cases, fathers were the key decision makers concerning where the mother gave birth from and whether care was sought for sick newborns. Similar findings were observed in a study in Eastern Uganda [19] and in Zambia [24]. We think this could be related to the financial implications of the decision to seek care outside the home [25]. Indeed, previous research has highlighted the role of husbands in acquiring transport, money, and other instrumental support needed in seeking care for pregnant women and their newborns [26,27,28]. Our findings differ from studies in Ethiopia and Ghana, which found that grandmothers played a key role in deciding where a woman should give birth [26,29,30]. Decision makers vary across different places, since decision making is socially and culturally constructed, and this could explain this difference [21]. The difference could result from differences in the respect given to grandmothers in different countries, or the ability of grandmothers to pay for the health care services of their daughters/daughters-in-law. In addition, the difference could be explained by the difference in study design and sampling methods used in these studies. The studies in Ethiopia and Ghana used a qualitative design, with nonrandom sampling (purposeful sampling), whereas we used a quantitative design with random sampling.

Grandmothers most commonly performed the first neonatal bath, whereas traditional birth attendants most commonly applied substances to the umbilical cord of the child in the period immediately after birth. In the period immediately after birth, mothers are (or perceived to be) exhausted [8], and the care of newborns is often delegated to older female relatives who are perceived to be knowledgeable and experienced [20,22]. This could explain why these older women were most commonly the ones who first bathed the baby, a finding that was also observed in communities in Nigeria, Tanzania, and Ethiopia [8]. As suspected, we found that grandmothers and traditional birth attendants were more involved in the postnatal care of first-time mothers, compared to mothers who had given birth before. First-time mothers are perceived to be least knowledgeable as regards newborn care [8]. Another reason that could explain greater grandmother involvement in the postnatal care of first-time mothers relates to the young age at which girls in Africa get pregnant [21]. Since the mothers are very young, societies have derived ways of training them in childcare, and central to this plan are the grandparents [21]. Young women often have to accept whatever decisions are made to avoid conflict [21].

Mothers were key decision makers relating to practices concerning breastfeeding. Since mothers spend the most time with the babies in the post-immediate period after birth, they are most likely to be the key decision makers concerning practices related to infant feeding. However, health workers were also common key decision makers in breastfeeding practices. Similar findings were seen in Malawi, where mothers were the most often-cited decision makers in breastfeeding, but grandmothers were also involved [11]. A study conducted in the same area showed that involvement of significant others in breastfeeding decisions was a predictor of early initiation of breastfeeding [31]. These findings from Uganda are in broad agreement with similar literature from Ethiopia, Tanzania, and Nigeria [8], and this suggests that the findings might be cross-cultural.

Regarding the decision on place of birth, mothers earning money and more educated mothers were more likely to decide on the place of birth. Similar findings have been observed in Ghana [32], and this suggests that decision-making could be a good measure of women empowerment [33]. Regarding the decision to practice timely breastfeeding initiation, older mothers and mothers in urban areas were more likely to decide on when to initiate breastfeeding. This implies that when designing interventions in rural areas, other people key in deciding on breastfeeding initiation like health workers need to be involved. Interventions that seek to promote health facility births need to involve husbands, and interventions to promote delayed bathing of the newborn need to involve grandparents.

In this study, we observed that men were not commonly involved in the selected newborn care practices, apart from deciding the place of birth and seeking care for sick newborns. Prior research has shown that improved male involvement is associated with improved maternal and newborn health through the promotion of skilled birth attendance, postpartum care, complications preparedness, and maternal nutrition [34]. As such, men need to be encouraged to have more involvement in newborn care, especially in being direct actors in newborn care practices. It has been reported that cultural perceptions hinder men from participating in the immediate care of newborns, as this period is gendered as feminine [25]. This could explain the absence of male involvement in this period, and campaigns aimed at encouraging male involvement need to address that issue.

This study had some limitations; the long recall period (11 months on average) could have introduced a measurement/information bias. We used this long recall window to enable us to obtain sufficient mothers using multistage sampling [35]. To minimize the recall limitation, we asked questions relating to only the most recent pregnancy and childbirth. We acknowledge that decision-making is a complex process involving multiple parties at different levels, and the utilization of a more detailed framework in data collection would have been ideal. Such a framework would enable us to address joint decision making, power dynamics, willingness or unwillingness of mothers to make decisions, changing trends of decision making over time, and influence of urbanization/globalization on decision making. It can, therefore, be argued that we oversimplified a very complex process. The role of different actors in childcare is rooted in larger sociocultural constructs [21] and is expected to vary from one place to another. This study was carried out in the Lira district, located in post-conflict Northern Uganda, and the findings reported can only be generalized to similar settings. We did not explore various aspects of the decision-making process.

## 5. Conclusions

Fathers, grandmothers, health-workers, and traditional birth attendants were influential in newborn care. Programs that aim to promote newborn care, especially programs that encourage facility births, and immediate newborn care, need to involve persons other than the mother; particularly husbands and grandmothers. Health workers should be involved in programs that promote timely initiation of breastfeeding, especially in rural areas. This study also highlights the importance of context-specific formative research prior to interventional studies being rolled out, to ensure that interventions are directed at the right people.

## Figures and Tables

**Table 1:** Baseline characteristics of mothers surveyed in the Lira district, Northern Uganda.

	Participants (*n* = 930) *n* (%)
Mother age	
≤19	157 (16.9)
20–35	686 (73.8)
>35	87 (09.4)
Mothers education	
None	102 (11.0)
Primary	729 (78.4)
Secondary	83 (08.9)
Tertiary	16 (01.7)
Paternal education	
None	24 (02.8)
Primary	524 (61.4)
Secondary	228 (26.7)
Tertiary	77 (09.0)
Marital status	
Single	77 (08.3)
Married	853 (91.7)
Mother formally employed	
No	601 (64.6)
Yes	329 (35.4)
Parity	
1	227 (24.4)
2–3	298 (32.0)
>4	405 (43.6)
Residence	
Rural	589 (63.3)
Urban	341 (36.7)
Place of birth	
Health facility	622 (66.9)
Home	308 (33.1)

**Table 2:** Table showing key decision makers and actors in newborn care in the Lira district, Northern Uganda.

Variable	All Births *n* = 930		Home Births *n* = 308	Health Facility Births *n* = 622	*p-*Value * (X^2^) ^†^
	*n* (%)	95%CI of %	*n* (%)	*n* (%)	
Decide birthplace					0.022 (3.933)
Father	505 (54.3)	49.4–59.2	148 (48.1)	357 (57.4)	
Mother	289 (31.1)	27.5–34.9	121 (39.3)	168 (27.0)	
Grandmother	94 (10.1)	8.2–12.4	27 (8.8)	67 (10.8)	
Others	42 (4.5)	3.4–6.0	12 (3.9)	30 (4.8)	
Dried baby immediately after birth					<0.001 (407.3)
Health worker	561 (63.8)	55.7–71.1	4 (1.4)	557 (95.1)	
TBA	238 (27.1)	20.5–34.8	235 (79.9)	3 (0.5)	
Others	81 (9.2)	7.2–11.7	55 (18.7)	26 (4.4)	
Conducted first bath					<0.001 (8.906)
Grandmother	516 (55.5)	51.9–59.1	142 (46.1)	374 (60.1)	
Mother	165 (17.7)	15.4–20.3	54 (17.5)	111 (17.9)	
Others	249 (26.8)	23.2–30.7	112 (36.4)	137 (22.0)	
Decide breastfeeding initiation					<0.001 (57.87)
Mother	499 (53.7)	49.8–57.5	171 (55.5)	328 (52.7)	
Health worker	239 (25.7)	22.1–29.7	9 (2.9)	230 (37.0)	
Grandmother	92 (9.9)	8.4–11.6	42 (13.6)	50 (8.0)	
TBA	77 (2.5)	5.8–11.7	77 (25.0)	0 (0.0)	
Others	23 (2.5)	1.6–3.7	9 (2.9)	14 (2.3)	
Decide what to do with initial breast milk					<0.001 (70.28)
Mother	534 (57.4)	54.5–60.3	175 (56.8)	359 (57.7)	
Health worker	207 (22.3)	19.8–24.9	12 (3.9)	195 (31.4)	
Grandmother	93 (10.0)	8.2–12.1	35 (11.4)	58 (9.3)	
Others	96 (10.3)	7.6–13.9	86 (27.9)	10 (1.6)	
Decide whether or not to practice bottle-feeding					<0.001 (8.184)
Mother	684 (73.6)	70.3–76.5	241(78.3)	443 (71.2)	
Health worker	90 (9.7)	7.5–12.3	10 (3.3)	80 (12.9)	
Grandmother	57 (6.1)	4.6–8.2	19 (6.2)	38 (6.1)	
Others	99 (10.7)	8.8–12.8	38 (12.3)	61 (9.8)	
Applied substances to the umbilical cord					<0.001 (12.00)
TBA	17 (28.8)	19.4–40.5	17 (53.1)	0 (0)	
Health Worker	16 (27.1)	16.0–42.1	0 (0)	16 (59.3)	
Mother	15 (25.4)	16.9–36.4	9 (28.1)	6 (22.2)	
Others	11 (18.6)	10.5–31.0	6 (18.8)	5 (18.5)	
Decide care seeking for sick newborn					0.688 (0.370)
Father	92 (47.7)	41.8–53.7	27(42.9)	65 (50.0)	
Mother	88 (45.6)	39.0–52.4	31(49.2)	57 (43.9)	
Others	13 (6.7)	3.9–11.5	5(7.9)	8 (6.2)	

TBA: Traditional birth attendants; *p*-value *: Pearson’s chi-squared test; (X^2^) ^†^: Chi-squared test statistic.

**Table 3:** Table showing key decision makers and actors in newborn care in Lira district, Northern Uganda.

	Rural Births *n* = 589 *n* (%)	Urban Births *n* = 341 *n* (%)	*p*-Value * (X^2^) ^†^	First-Time Mothers *n* = 227 *n* (%)	Mothers with Previous Birth *n* = 703 *n* (%)	*p*-Value * (X^2^) ^†^
Decide birthplace			0.498 (0.766)			<0.001(21.03)
Father	311 (52.8)	194 (56.9)		114 (50.2)	391 (55.6)	
Mother	194 (32.9)	95 (27.9)		51 (22.5)	238 (33.9)	
Grandmother	59 (10.0)	35 (10.3)		48 (21.2)	46 (6.5)	
Others	25 (4.2)	17 (5.0)		14 (6.2)	28 (4.0)	
Dried baby immediately after birth			<0.001 (23.16)			<0.001(10.62)
Health worker	302 (53.6)	259 (81.7)		160 (74.8)	401 (60.2)	
TBA	199 (35.4)	39 (12.3)		44 (20.6)	194 (29.1)	
Others	62 (11.0)	19 (6.0)		10 (4.7)	71 (10.7)	
Conducted first bath			0.090 (2.591)			<0.001(32.21)
Grandmother	344 (58.4)	172 (50.4)		175 (77.1)	341 (48.5)	
Mother	100 (17.0)	65 (19.1)		9 (4.0)	156 (22.2)	
Others	145 (24.6)	104 (30.5)		43 (18.9)	206 (29.3)	
Decide breastfeeding initiation			<0.001 (5.813)			<0.001(8.116)
Mother	303 (51.4)	196 (57.5)		91 (40.1)	408 (58.0)	
Health worker	144 (24.5)	95 (27.9)		75 (33.0)	164 (23.3)	
Grandmother	61 (10.4)	31 (9.1)		39 (17.2)	53 (75.4)	
TBA	68 (11.5)	9 (2.6)		16 (7.1)	61 (8.7)	
Others	13 (2.2)	10 (2.9)		6 (2.6)	17 (2.4)	
Decided what to do with initial breast milk			<0.001 (7.54)			<0.001(9.027)
Mother	321 (54.5)	213 (62.5)		87 (38.3)	447 (63.6)	
Health worker	125 (21.2)	82 (24.1)		66 (29.1)	141 (20.1)	
Grandmother	67 (11.4)	26 (7.6)		44 (19.4)	49 (7.0)	
Others	76 (12.9)	20 (5.9)		30 (13.2)	66 (9.4)	
Decide whether or not to practice bottle-feeding			0.318 (1.189)			<0.001(10.27)
Mother	441 (74.9)	243 (71.3)		150 (66.1)	534 (76.0)	
Health worker	49 (8.3)	41 (12.0)		22 (9.7)	68 (9.7)	
Grandmother	36 (6.1)	21 (6.2)		31 (13.7)	26 (3.7)	
Others	63 (10.7)	36 (10.6)		24 (10.6)	75 (10.7)	
Applied substances to the umbilical cord			0.112 (2.099)			0.179(1.708)
TBA	15 (37.5)	2 (10.5)		9 (40.9)	8 (21.6)	
Health Worker	8 (20.0)	8 (42.1)		7 (31.8)	9 (24.3)	
Mother	10 (25.0)	5 (26.3)		2 (9.1)	13 (35.1)	
Others	7 (17.5)	4 (21.1)		4 (18.2)	7 (18.9)	
Decide care seeking for sick newborn			0.240 (1.468)			0.003 (6.543)
Father	58 (43.6)	34 (56.7)		29 (53.7)	63 (45.3)	
Mother	66 (49.6)	22 (36.7)		16 (29.6)	72 (51.8)	
Others	9 (6.8)	4 (6.7)		9 (16.7)	4 (2.9)	

*p*-value *: Pearson’s chi-squared test; (X^2^) ^†^: Chi-squared test statistic.

**Table 4:** Factors associated with mothers being key decision makers on place of birth in the Lira district, Northern Uganda.

	Bi-Variable *n* = 930 OR (95% CI)	*p-*Value	Multivariable *n* = 930 AOR (95% CI)
Mother age			
≤19	1		1
20–35	1.6 (1.1, 2.4)	0.018	1.1 (0.60, 2.0)
>35	1.3 (1.3, 4.3)	0.008	1.3 (0.58, 3.1)
Mother’s Wealth index			
Lowest	1		1
2	0.89 (0.58, 1.4)	0.590	0.81 (0.51, 1.3)
3	0.90 (0.57, 1.4)	0.639	0.74 (0.47, 1.2)
4	0.95 (0.64, 1.4)	0.809	0.77 (0.50, 1.2)
Highest	0.59 (0.38, 0.93)	0.024	0.45 (0.26, 0.75)
Mothers education			
None	1		1
Primary	0.84 (0.52, 1.34)	0.451	1.2 (0.73, 1.9)
Secondary	1.0 (0.57, 1.86)	0.925	1.9 (1.0, 3.6)
Tertiary	0.44 (0.12, 1.6)	0.200	0.84 (0.21, 3.4)
Fathers education			-
None	1		
Primary	0.39 (0.19, 0.80)	0.012	
Secondary	0.46 (0.22, 0.97)	0.042	
Tertiary	0.33 (0.13, 0.85)	0.023	
Marital status (living with partner)			-
Single	1		
Married	0.38 (0.23, 0.63)	0.001	
Parity			
1	1		1
2–3	1.4 (0.94, 2.0)	0.101	1.3 (0.85, 2.1)
>4	2.1 (1.5, 3.0)	<0.001	2.1 (1.2, 3.8)
Residence			-
Urban	1		
Rural	1.3 (0.90, 1.8)	0.167	
Mother formally employed			
No	1		1
Yes	1.9 (1.4, 2.7)	<0.001	2.0 (1.5, 2.9)

**Table 5:** Factors associated with mothers being the key decision makers on when to initiate breastfeeding in the Lira district, Northern Uganda.

	Bi-Variable *n* = 930 OR (95% CI)	*p*-Value	Multivariable *n* = 930 AOR (95% CI)
Mother age			
≤19	1		1
20–24	2.0 (1.3–3.2)	0.003	1.8 (1.1–3.0)
25–29	2.5 (1.6–3.9)	0.000	1.9 (1.1–3.4)
30–34	3.5 (2.1–6.0)	0.000	2.8 (1.3–6.1)
≥35	3.9 (2.3–6.8)	0.000	3.1 (1.5–6.4)
Mother’s Wealth index			–
1 (Lowest)	1		
2	1.1 (0.72–1.6)	0.777	
3	1.3 (0.89–1.8)	0.171	
4	1.0 (0.68–1.5)	0.999	
5 (Highest)	1.2 (0.82–1.9)	0.289	
Mothers education			
None	1		1
Primary	0.90 (0.65–1.2)	0.493	1.3 (0.87–1.9)
Secondary	0.93 (0.46–1.9)	0.848	1.3 (0.63–2.7)
Tertiary	1.0 (0.35–2.9)	0.977	1.4 (0.40–4.7)
Fathers education			–
None	1		
Primary	1.2 (0.48–2.9)	0.704	
Secondary	1.3 (0.49–3.4)	0.606	
Tertiary	1.1 (0.44–3.0)	0.783	
Marital status (living with partner)			
Single	1		1
Married	1.5 (0.82–2.8)	0.176	1.2 (0.67–2.1)
Parity			
1	1	1	1
2–3	1.6 (1.2–2.2)	0.002	1.2 (0.79–1.7)
>4	2.5 (1.8–3.4)	<0.001	1.3 (0.76–2.4)
Residence			
Urban	1	1	1
Rural	0.78 (0.59–1.1)	0.095	0.72 (0.52–0.99)
Mother formally employed			
No	1		1
Yes	1.1 (0.87–1.5)	0.349	1.1 (0.85–1.4)
Place of Birth			
Home	1		1
Health facility	0.89 (0.68–1.2)	0.414	0.86 (0.64–1.2)

## Appendix A

**Table A1:** Table showing a detailed list of key decision makers and actors in newborn care in the Lira district, Northern Uganda.

Variable	*n* (%)	95% CI of %
Decide birthplace		
Mother of baby	289 (31.08)	27.53–34.86
Father of baby	505 (54.30)	49.35–59.17
* Mother-in-law	57 (6.13)	4.77–7.85
Sister	6 (0.65)	0.26–1.59
* Grandmother	37 (3.98)	2.79–5.64
Friend	2 (0.22)	–
TBA	4 (0.43)	0.16–1.13
Health worker	6 (0.65)	0.03–1.38
Other	24 (2.58)	1.73–3.82
Cut and tied the umbilical cord		
Mother of baby	13 (1.40)	0.78–2.50
Father of baby	2 (0.22)	–
Mother-in-law	16 (1.72)	1.03–2.86
Sister	3 (0.32)	–
Grandmother	12 (1.29)	0.76–2.18
Friend	1 (0.11)	–
TBA	257 (27.63)	20.89–35.58
Health worker	617 (66.34)	58.02–73.77
Other	9 (0.97)	0.46–2.02
Applied substances to the umbilical cord		
Mother of baby	15 (25.42)	16.88–36.40
Father of baby	1 (1.69)	0.20–12.68
Mother-in-law	4 (6.78)	2.81–15.45
Sister	1 (1.69)	0.2–12.68
Grandmother	5 (8.47)	2.97–21.85
TBA	17 (28.81)	19.39–40.51
Health worker	16 (27.12)	15.99–42.12
First bathed the baby immediately after birth		
Mother of baby	165 (17.74)	15.43–20.32
Father of baby	7 (0.75)	0.38–1.49
Mother-in-law	340 (36.56)	33.58–39.65
Sister	46 (4.95)	3.50–6.95
Grandmother	176 (18.92)	15.97–22.29
Friend	24 (2.58)	1.50–4.41
TBA	76 (8.17)	5.20–12.62
Health worker	13 (1.40)	0.78–2.50
Other	83 (8.92)	6.90–11.48
Decide breastfeeding initiation		
Mother of baby	499 (53.66)	49.79–57.48
Mother-in-law	57 (6.13)	4.91–7.63
Sister	5 (0.54)	0.19–1.50
Grandmother	35 (3.76)	2.58–5.47
Friend	1 (0.11)	–
TBA	77 (8.28)	5.78–11.72
Health worker	239 (25.70)	22.08–29.68
Other	17 (1.83)	1.21–2.76
Decide what to do with initial breast milk		
Mother of baby	534 (57.4)	54.49–60.30
Mother in Law	59 (6.34)	5.00–8.02
Sister	4 (0.43)	-
Grandmother	34 (3.66)	2.56-5.19
Friend	2 (0.22)	-
TBA	83 (8.92)	6.24–12.61
Health Worker	207 (22.26)	19.84–24.88
Other	7 (0.75)	0.38–1.49
Decide whether or not to practice bottle-feeding		
Mother of baby	684 (73.55)	70.33–76.54
Father of baby	3 (0.32)	–
Mother-in-law	37 (3.98)	2.90–5.44
Sister	2 (0.22)	–
Grandmother	20 (2.15)	1.31–3.52
TBA	15 (1.61)	0.9–2.87
Health worker	90 (9.68)	7.54–12.34
Other	79 (8.49)	6.81–10.55
Decide care seeking for ill newborn		
(For those who had sick newborns and sought care)		
Mother of baby	88 (45.60)	38.97–52.38
Father of baby	92 (47.67)	41.76–53.65
Mother-in-law	2 (1.04)	0.26–4.02
Grandmother	7 (3.63)	1.76–7.33
Other	4 (2.07)	0.78–5.41

* Grandmother refers to the mother of the mother of the baby. * Mother-in-law refers to the mother of the father of the baby.
